# Focus on patient perspectives in climate action policies for healthcare. A German survey analysis on what patients are willing to do

**DOI:** 10.3389/fpubh.2024.1477313

**Published:** 2024-11-26

**Authors:** Florian Scholz, Nikolaus Börner, Sophie Anne Schust, Josefine Schardey, Florian Kühn, Bernhard Renz, Martin Angele, Jens Werner, Markus Guba, Sven Jacob

**Affiliations:** Department of General, Visceral and Transplantation Surgery, Ludwig-Maximilians-University, Munich, Germany

**Keywords:** climate change, healthcare, patient perspective, advocacy, policy, implementing change

## Abstract

**Background:**

The healthcare sector, while dedicated to improving health, paradoxically contributes significantly to global carbon emissions, accounting for approximately 4.9% of global emissions. Despite growing public concern about climate change, few studies have explored patients’ awareness and attitudes toward the environmental impact of healthcare. This study aims to assess patients’ perspectives on climate change and the sustainability of healthcare practices.

**Methods:**

A cross-sectional survey was conducted at Ludwig-Maximilians-University (LMU) Hospital in Munich, Germany. Patients were invited to participate in a voluntary, anonymous online survey via strategically placed QR codes throughout the hospital. The survey explored patients’ demographic information, environmental awareness, attitudes toward climate-friendly practices in healthcare, and willingness to support sustainable initiatives. Descriptive statistics and regression analyses were used to analyze the data.

**Results:**

A total of 399 patients completed the survey (87% completion rate). The majority of respondents (92.3%) were aware of climate change, and 82.7% reported prioritizing climate-friendly practices in their personal lives. However, 55.9% of respondents were unaware of the healthcare system’s contribution to carbon emissions, and only 18.3% knew about hospitals’ climate impact. Despite this, 88.2% of respondents supported environmentally friendly initiatives in hospitals, and 86.5% were open to sustainable alternatives, provided that quality standards were maintained. Participants expressed significant interest in knowing the environmental impact of their treatments, with 63.2% in favor of a Nutri-Score-like system that would display the carbon footprint of medical procedures. Among those, 54.4% indicated that such a system would influence their choice of treatment. Similarly, 62.2% of respondents were interested in knowing the environmental impact of their medications, with 65% reporting that this information would affect their medication choices. A notable proportion of patients (66.2%) indicated willingness to support sustainable healthcare through shorter hospital stays and increased follow-up visits, while 35.8% were open to paying a CO_2_ compensation fee for their treatments. However, 81% were unwilling to pay higher insurance premiums to support environmentally friendly practices in hospitals. Regression analyses revealed that older age groups and having children were positively associated with environmental awareness (*p* < 0.05). However, factors such as gender, education level, relationship status, and illness severity did not significantly impact environmental attitudes. There was a significant correlation between patients’ environmental friendliness and their readiness to take climate-protective actions (*p* < 0.001).

**Conclusion:**

The study highlights a gap between patients’ environmental awareness and their knowledge of healthcare’s carbon footprint. While patients are generally supportive of sustainable practices in healthcare, their willingness to act diminishes when personal costs or discomfort are involved. A coordinated approach involving policy changes, patient education, and market innovations is essential to promote sustainable practices in healthcare without compromising patient care quality. Further research is needed to explore strategies for bridging the gap between environmental awareness and action in healthcare settings.

## 1 Introduction

In medicine, a “circulus vitiosus” describes a pathophysiological process in which two or more pathological bodily functions influence each other in a way of positive enhancement and therefore sustain a disease (1). This concept fittingly characterizes the correlation between the healthcare sector and climate change. The healthcare sector, which aims to protect and improve health, paradoxically contributes to global warming, a major health threat. According to the Intergovernmental Panel on Climate Change, there is substantial evidence that human actions, primarily through the emission of greenhouse gasses, have undeniably led to global warming, resulting in a global surface temperature rise of 1.1°C above pre-industrial levels. The World Health Organization (WHO) has declared climate change the most significant global health threat of the 21st century (2, 3). Climate scientists have emphasized that immediate, concerted, and determined action against climate change is necessary (4).

Healthcare contributes approximately 4.9 percent to worldwide carbon emissions, exceeding the emissions of typical culprits such as aviation (up to 2.4%), shipping (1.7%), and approximately 50% higher than the automotive industry (5, 6). To put it abstractly: if healthcare were a country, it would rank as the fifth-largest carbon emitter worldwide (2, 7). These greenhouse gas emissions can be attributed to energy consumption, transportation, and the production, utilization, and disposal of products (7). Furthermore, the pharmaceutical industry significantly contributes through production and supply chains (5). It can thus be stated that healthcare significantly contributes to the progression of global warming and hence associated public health risks. While the scope of these emissions is well-documented, there remains a significant gap in translating this knowledge into actionable strategies within healthcare systems.

Surgical departments, including the need for anesthesia during operations, are the top carbon emitters in hospitals, followed by departments performing endoscopy and obstetrics. Studies have documented that operating theaters consume three to four times as much energy as other hospital areas, yet few have provided solutions beyond technological fixes, such as optimizing ventilation systems. The accumulated waste amounts to roughly 30% of hospitals’ waste. Furthermore, these waste products, potentially infectious, require energy-intensive disposal (3). Anesthetic gasses contribute largely to atmospheric pollution due to low metabolization rates and the absence of filter systems (8). Simultaneously there is a lack of robust frameworks or regulations governing their use and disposal. A systematic review on carbon emissions in hospitals, published by the Royal College of Surgeons, identified medical devices and consumables as the largest carbon hotspots (9). This includes manufacturing and, to a large extent, supply chains (10). Despite these findings, there is a lack of comprehensive initiatives within healthcare to reduce emissions, particularly in high-emitting departments.

As the healthcare sector expands to address health demands, it is imperative to detach this growth from its impact on the environment. Integrating climate considerations into various aspects of the healthcare sector is essential, such as digital health solutions, pandemic and climate preparedness, and sustainable efforts to achieve net-zero emissions. This includes climate education for the health workforce, incentivizing resilience and low-carbon operations through insurance schemes, and other actions to accelerate transformative change (7). Despite growing public concern about climate change and increased academic interest in associated health risks, few studies have assessed patients’ and physicians’ opinions on the health impact of climate change (11). A survey conducted by the New England Journal of Medicine (NEJM) on patients’ awareness showed that patients have little to no awareness of the health impacts of climate change (12). A similar level of awareness can be expected regarding the healthcare system’s impact on global climate (13). To date, there is a significant lack of studies quantifying this aspect.

With patients being the subjects of medical care and important stakeholders in the healthcare system, their involvement is vital to implementing climate action. Particularly in the context of climate change, the pressure on stakeholders to implement change is largely influenced by public discourse. However, concerning actions mitigating climate change, one might argue that stakeholders providing services to maintain health are unwilling to compromise. Thus far, patients have not been involved as partners in building climate-resistant healthcare systems (14). Even though patient participation, in general, is associated with better healthcare processes and patient health outcomes, reduced mortality, and lower healthcare costs (15). Despite the significance of the human element for climate initiatives, there is a lack of comprehensive understanding regarding the global population’s readiness to unite and combat climate change (4). This gap in understanding highlights an essential area of intervention where education and engagement could not only raise awareness but also foster patient-driven advocacy for sustainable healthcare practices.

While much of the current literature focuses on quantifying healthcare’s environmental impact, there is a pressing need for research that moves beyond description to critically analyze the barriers and opportunities for transformative change. The purpose of this survey study is to explore patients’ perspectives on the climate impact and support environmental initiatives in the healthcare setting. With this insight in mind, we can promote the execution of experimental studies with the goal of developing effective strategies for catalyzing change. This study can also serve as an educational tool in sensitizing patients toward the climate impact of the healthcare sector. Furthermore, sharing these findings would be crucial for garnering support, especially in efforts to gain political and industrial endorsement for adopting revised strategies aimed at reducing environmental impacts. This might involve increasing maintenance budgets or seeking funding for research initiatives.

## 2 Methods

### 2.1 Study design and objectives

The primary objective of this study was to examine patients’ awareness of CO_2_ emissions in the hospital and explore their willingness to participate in carbon footprint reduction. A voluntary, non-incentivized, anonymous cross-sectional survey of patients treated at the Ludwig-Maximilians-University (LMU) Hospital in Munich, Germany, was conducted. The sample population was defined through purposive sampling, aiming to capture a broad spectrum of patients from different wards and treatment areas within the hospital. The questionnaire was distributed to patients in various departments of the hospital. The participants included both out-patients and in-patients, who were undergoing different types of treatments, ranging from general surgery to internal medicine. Patients were randomly invited to participate by accessing an online survey via a QR code. This QR code was strategically distributed in printed form across various locations within both hospital campuses, including wards, outpatient areas, and emergency departments. The survey titled “Sustainability at the Hospital” consisted of 23 questions. It was created using LimeSurvey (LimeSurvey GmbH. LimeSurvey: An Open Source survey tool/LimeSurvey GmbH, Hamburg, Germany, http://www.limesurvey.org), and designed as a fully anonymous questionnaire with 46 items across four sections. The survey comprised both closed-ended and multiple-choice questions, using Likert scales for certain questions to measure the degree of agreement or experience (e.g., 1 = slightly sick, 5 = severely sick) as well as open questions.

The first section, “Personal Details and Illness,” gathered demographic data such as age, sex, and relationship status, alongside information on previous illnesses and details of the current hospital stay. The second section, “Environmental Information,” focused on the respondents’ general opinions on climate change, their carbon footprint, and climate protective actions in their daily lives and workplaces. The third section, “Environmental Protection in the Hospital,” sought patients’ opinions on the healthcare system’s contribution to climate change and potential actions to mitigate it. Finally, the “Final Statements” section assessed patients’ prior knowledge of the environmental impact of healthcare and provided some educational content.

**Figure 1:**
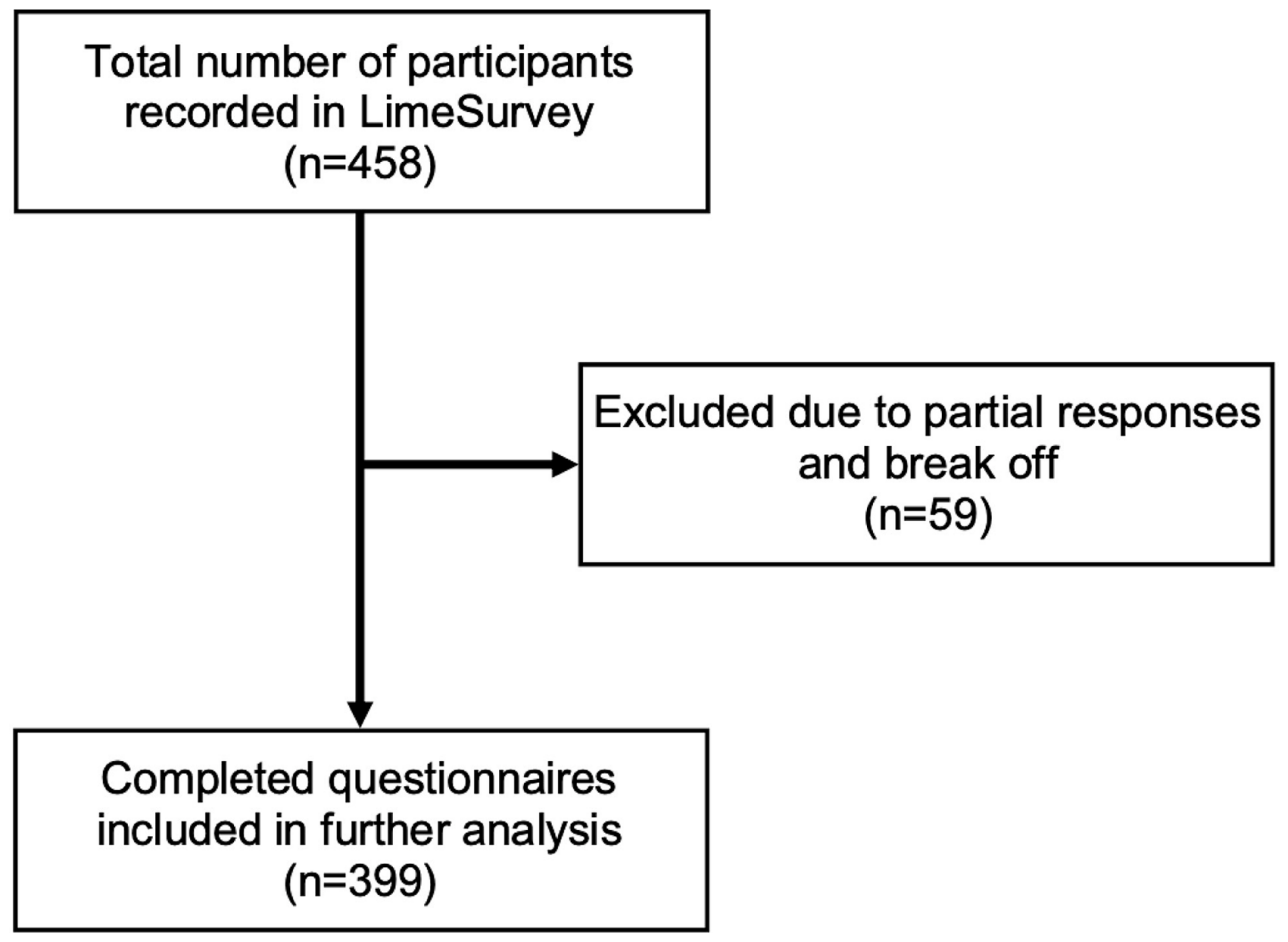
Final survey inclusion.

Participants were offered the choice to take the survey in either German or English. Only fully completed surveys were included in the final analysis (Figure 1). Premature termination of the survey was noted by the screen at which it was omitted at Table 1. The survey adhered to the AAPOR guidelines for survey studies and the CHERRIES guidelines were followed for clarity (16, 17).

**Table 1:** Survey participation and completion rates: overview of respondents and missing data.

Survey response		Count (%)
	Approached	458 (100)
	Completed	399 (87.1)
	Incomplete	59 (12.9)
Survey terminated on page	0	1 (0.2)
	1	23 (5.0)
	2	28 (6.1)
	3	7 (1.5)
	4	399 (87.1)
Missing values	None	399 (87.1)
	Yes	59 (12.9)

### 2.2 Data collection

The data collection process was carried out using an online survey, which participants accessed via printed QR codes. These QR codes were strategically placed in various locations within the hospital, including waiting rooms, outpatient departments, and different wards. Patients were also personally approached by hospital staff in waiting rooms and on the wards and were encouraged to participate.

The survey remained open for a total of 12 weeks, with the sampling period beginning upon the distribution of the QR codes. To ensure the integrity of the data, respondents were required to register with an email address, and only one survey per registered email was permitted. The survey system prevented participants from completing the questionnaire more than once with the same registration.

All collected data was stored anonymously on a secure server provided by LimeSurvey for the duration of the study, ensuring confidentiality. Participants were only able to complete the survey once per access session, and reminders to complete partially finished surveys were not sent. Incomplete submissions were automatically excluded from the final analysis. The full survey is available in Supplementary Data Sheet 1.

### 2.3 Statistical analysis

All statistical analyses were conducted using IBM SPSS Statistics version 29.0.2. Descriptive statistics were utilized to summarize demographic characteristics of the sample. To assess associations between categorical variables, chi-square tests were performed, and the significance level was set at *p* < 0.05. To investigate the relationships between predictor variables and outcome measures, both linear and multiple regression models were employed, with the dependent variable being environmental awareness. Independent variables included age, gender, relationship status, education level, number of children, and degree of illness.

Patient attitudes toward environmental protection and their readiness to act were assessed using a six-item Likert scale. The responses were analyzed to form a composite scale that represents overall environmental awareness and readiness to act, respectively, among the respondents. The reliability of the scale was tested using Cronbach’s alpha, yielding a value of 0.824, indicating strong internal consistency. Each item’s corrected item-total correlation supported the scale’s robustness. Item-level analysis showed that deleting any item would not significantly improve reliability, confirming the scale’s suitability for measuring environmental awareness and readiness to act in this patient population (47).

In the multiple regression models, environmental awareness was the dependent variable, and the key predictors were age group, gender, relationship status, education level, number of children, and degree of illness. The model aimed to evaluate the impact of these factors while controlling for potential confounders. Additionally, to assess the relationship between environmental awareness and willingness to take climate-protective actions, a separate linear regression was performed with willingness to act as the dependent variable and environmental awareness as the key predictor.

Diagnostic analyses, including tests for multicollinearity, heteroscedasticity, and outliers, were conducted to evaluate the goodness of fit of the regression models. No significant violations of the regression assumptions were identified, indicating that the models provided robust estimates. The overall fit of the models was assessed using ANOVA and R-squared values. For the environmental awareness model, the R-squared value was 0.223, and for the willingness-to-act model, it was 0.469, indicating the percentage of variance explained by the predictors in each model. The results of the regression analyses are further detailed in the results section.

Pearson’s correlation coefficient was applied to evaluate relationships between environmental awareness and readiness to engage in pro-environmental behaviors. Additionally, an analysis of variance (ANOVA) was conducted to assess differences in environmental awareness across groups. The positive correlation between environmental awareness and actions was further quantified using eta squared (*η*^2^ = 0.576, *p* < 0.001).

### 2.4 Ethics statement

The study underwent review by the local ethics board and the data protection officer. Given that it involved an anonymous questionnaire, ethical approval was waived by the Ethics Committee of Ludwig-Maximilian University (LMU) Munich (Survey application ID 1896, chaired by Gerhard Meyer, LMU Munich, Pettenkoferstr. 8a, 80,336 München, Germany). Participants were duly informed that submitting the questionnaire implied consent to participate in the study and its procedures.

## 3 Results

### 3.1 Study demographics

A total of 458 individuals initiated the survey protocol, of which 87% completed the survey and were included in further analysis (*n* = 399). Completion of the survey was monitored by on which page the survey was terminated. A table of survey completion is shown in Table 1 and final inclusion is depicted in Figure 1.

**Figure 2:**
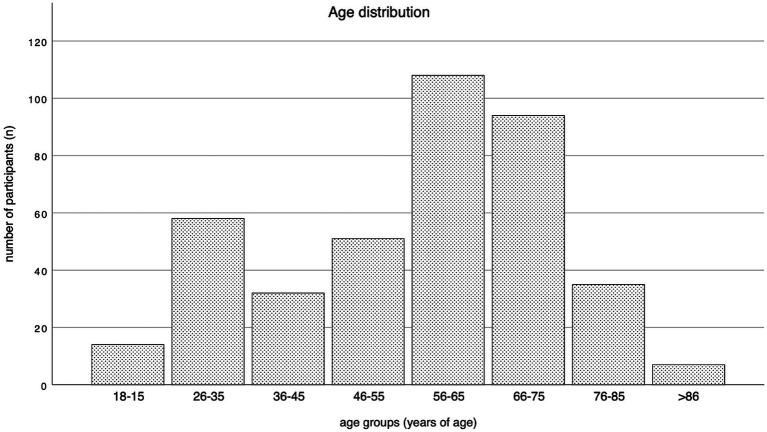
Age distribution by age groups.

Approximately twice as many men responded to the survey (*n* = 259, 64%) compared to women (*n* = 138, 36%). The age distribution of 56–65 years and 66–75 years comprised the largest proportion of the cohort. The complete age distribution of respondents is shown in Figure 2, categorized by age groups. Regarding educational attainment, 31% of respondents indicated that they had obtained a university degree (*n* = 125), while 25% (*n* = 102) had completed an apprenticeship or acquired a college degree. For 15% of respondents, completing high school represented the highest level of formal education attained (*n* = 62). An overview of Patients demographics is provided in Table 2.

**Table 2:** Respondents’ demographic characteristics were assessed through initial questions on demographic data.

Patient demographics		Count (%)
Language	German	387 (97)
	English	12 (3)
Gender	Male	365 (91.5)
	Female	34 (8.5)
	Divers	1 (1)
Age-group	18–25	14 (4)
	26–35	58 (15)
	36–45	32 (8)
	46–55	51 (13)
	56–65	108 (27)
	66–75	94 (24)
	76–85	35 (9)
	>86	7 (2)
Relationship status	Married/civil union	234 (59)
	Single	63 (16)
	Partnership	53 (13)
	Divorced	30 (8)
	Widowed	19 (5)
Children	Yes	275 (69)
	No	330 (82.7)
Educational qualification	Middle School diploma	45 (11.3)
	Secondary school	63 (15.8)
	General university entrance qualification	62 (15.5)
	Completed vocational training/University of Applied Sciences degree	102 (25.6)
	University degree/Doctorate/Habilitation	125 (31.3)
	No diploma	2 (0.5)
Place of residence	Germany	396 (99.3)
	Abroad	3 (0.8)
	Bavaria	379 (95)
	Other states	17 (4.3)

Age, gender, relationship status, number of children were assessed by predefined answers. Educational attainment and place of residence were asked as open questions and given results analyzed and categorized.

### 3.2 Medical characteristics

At the time of responding to the questionnaire, 84% of participants were undergoing inpatient treatment (*n* = 336), while 63 participants responded as outpatients (16%). The largest fraction of respondents received treatment in the urology department (33.6%), followed by trauma/orthopedics (25.3%), internal medicine (24.8%) and the general surgery department (8%, *n* = 32). The majority of respondents (52.1%, *n* = 208) reported having no or mild prior diseases, while 26.3% (*n* = 105) reported having severe preexisting conditions. Most respondents underwent a surgical procedure or had one scheduled during their stay (82%, *n* = 329). 31% had been diagnosed with cancer at the time of responding, of whom 39% (*n* = 53) were currently undergoing treatment. Additionally, 41% of respondents had experienced cancer in the past. Concerning the length of hospital stay, 47.1% of patients expected to stay for 1–5 days, while 31.6% (*n* = 125) reported an expected duration of stay between 5 and 10 days. Further medical characteristics are depicted in Table 3.

**Table 3:** Respondents’ medical characteristics were assessed through questions covering the nature of the hospital stay, current health status, and medication use.

Respondents medical characteristics		Count (%)
Nature of the stay	Outpatient	63 (15.8)
	Inpatient	336 (84.2)
Admitting department	Internal medicine	99 (24.8)
	Orthopedics/trauma surgery	101 (25.3)
	General surgery	32 (8.0)
	Neurosurgery	8 (2.0)
	Urology	134 (33.6)
	Gynecology/obstetrics	3 (0.8)
	ENT	4 (1.0)
	Plastic surgery	3 (0.8)
	Others	15 (3.8)
Degree of Illness (1 = slightly ill to 5 = very ill)	1	43 (10.8)
	2	73 (18.3)
	3	133 (33.3)
	4	96 (24.1)
	5	54 (13.5)
Prior Illness	No prior illness	132 (33.1)
	Mild prior illness	76 (19.0)
	Moderate prior illness	86 (21.6)
	Rather severe prior illness	66 (16.5)
	Severe prior illness	39 (9.8)
Performance capability	Normal performance, no complaints, no manifest illnesses	63 (15.8)
	Normal performance, minimal symptoms of illness	93 (23.3)
	Normal performance with exertion, minor symptoms of illness	78 (19.5)
	Limited performance, unable to work, self-care	77 (19.3)
	Limited performance, occasionally need help from others	40 (10.0)
	Limited performance, need nursing and medical care, not permanently bedridden	32 (8.0)
	Bedridden, special care required	10 (2.5)
	Severely ill, hospital care required	6 (1.5)
Surgical procedure	None	70 (17.5)
	Scheduled	84 (21.1)
	Already carried out	245 (61.4)
Tumor disease	Yes	124 (31.1)
	No	263 (65.9)
	Not sure	12 (3.0)
Tumor disease, currently treated	Yes	60 (15.0)
	No	329 (82.5)
	Unsure	10 (2.5)
Tumor disease in the past	Yes	75 (18.8)
	No	321 (80.5)
	Not sure	3 (0.8)
Current medication	No medication	115 (28.8)
	Less than 5 medications	190 (47.6)
	More than 5 medications	69 (17.3)
	More than 10 medications	25 (6.3)
Expected length of stay	1–5 days	188 (47.1)
	5–10 days	126 (31.6)
	10–15 days	47 (11.8)
	More than 15 days	38 (9.5)

The response items were predefined, and the admitting department was specified by the respondents. The frequencies of selected items are presented in absolute numbers and as percentages of the total cohort.

### 3.3 General attitude toward the environment

**Figure 3:**
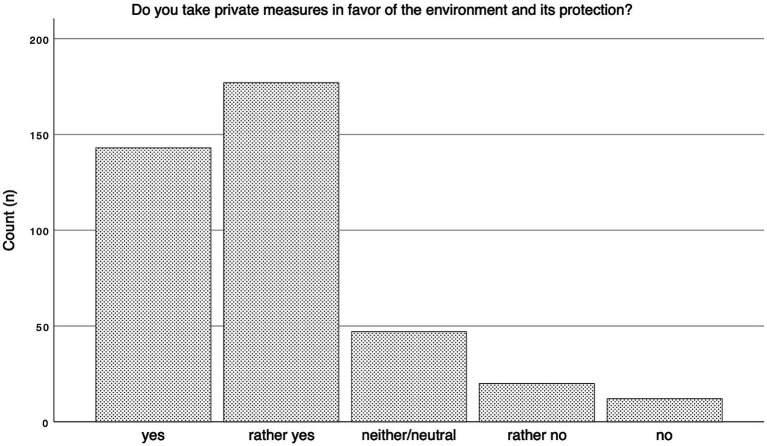
Answer frequency environmental action.

90.2% of respondents stated that they generally care about the environment (*n* = 360). Additionally, 92.3% (*n* = 368) of participants reported awareness of climate change, with 82.7% (*n* = 330) stating that climate-friendly practices had a high priority in their private lives. Figure 3 shows the frequency in absolute numbers of respondents’ answers to whether they take private measures in favor of the environment and its protection. Using a Likert scale, respondents’ attitudes toward environmental protection were assessed through six questions, resulting in a scale representative of patients’ environmental awareness. Sufficient intervariable consistency and reliability were documented using Cronbach’s alpha (0.794). The resulting scale revealed a mean environmental friendliness of 4.25 on a scale of one to five and was utilized in further regression analysis. Further Participants were asked about their engagement in various environmental protection measures during everyday life. The frequency of responses is depicted in Table 4.

**Table 4:** Respondents were asked to indicate their engagement in everyday environmental protection measures from a provided list.

Engagement in environmental protection measures		Count (%)
Avoiding waste (e.g., through “unpackaged stores,” shopping at the market with reusable bags, etc.)	Yes	234 (58.6)
	No	165 (41.4)
Waste separation	Yes	365 (91.5)
	No	34 (8.5)
CO_2_-neutral travel by bicycle/public transport/electric car or similar	Yes	196 (49.1)
	No	203 (50.9)
Saving water (e.g., turning off the water when brushing teeth)	Yes	311 (77.9)
	No	88 (22.1)
Showering instead of bathing	Yes	330 (82.7)
	No	69 (17.3)
Air-dry hair instead of blow-drying	Yes	204 (51.1)
	No	195 (48.9)
Avoidance of animal products	Yes	44 (11.0)
	No	355 (89.0)
Purchase of food from controlled organic cultivation	Yes	187 (46.9)
	No	212 (53.1)
Purchase of regional food	Yes	283 (70.9)
	No	116 (29.1)
Avoid batteries/use mains-powered or battery-free devices	Yes	113 (28.3)
	No	286 (71.7)
No measures	Yes	8 (2.0)
	No	391 (98.0)

The frequencies of selected items are presented in absolute numbers and as percentages of the total cohort.

**Figure 4:**
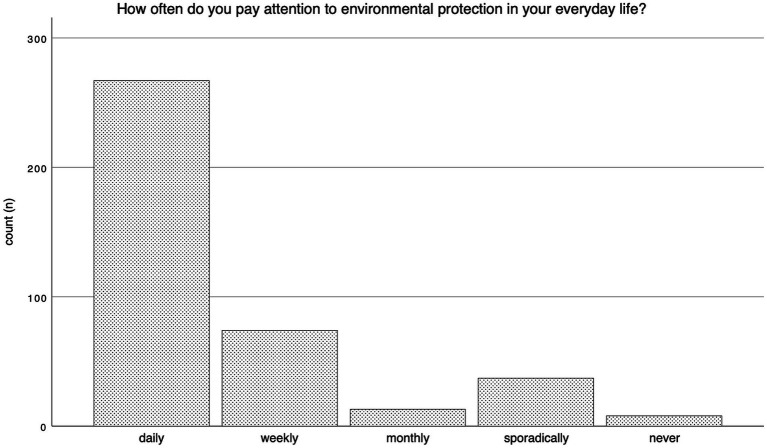
Frequency of environmental protective measures.

**Figure 5:**
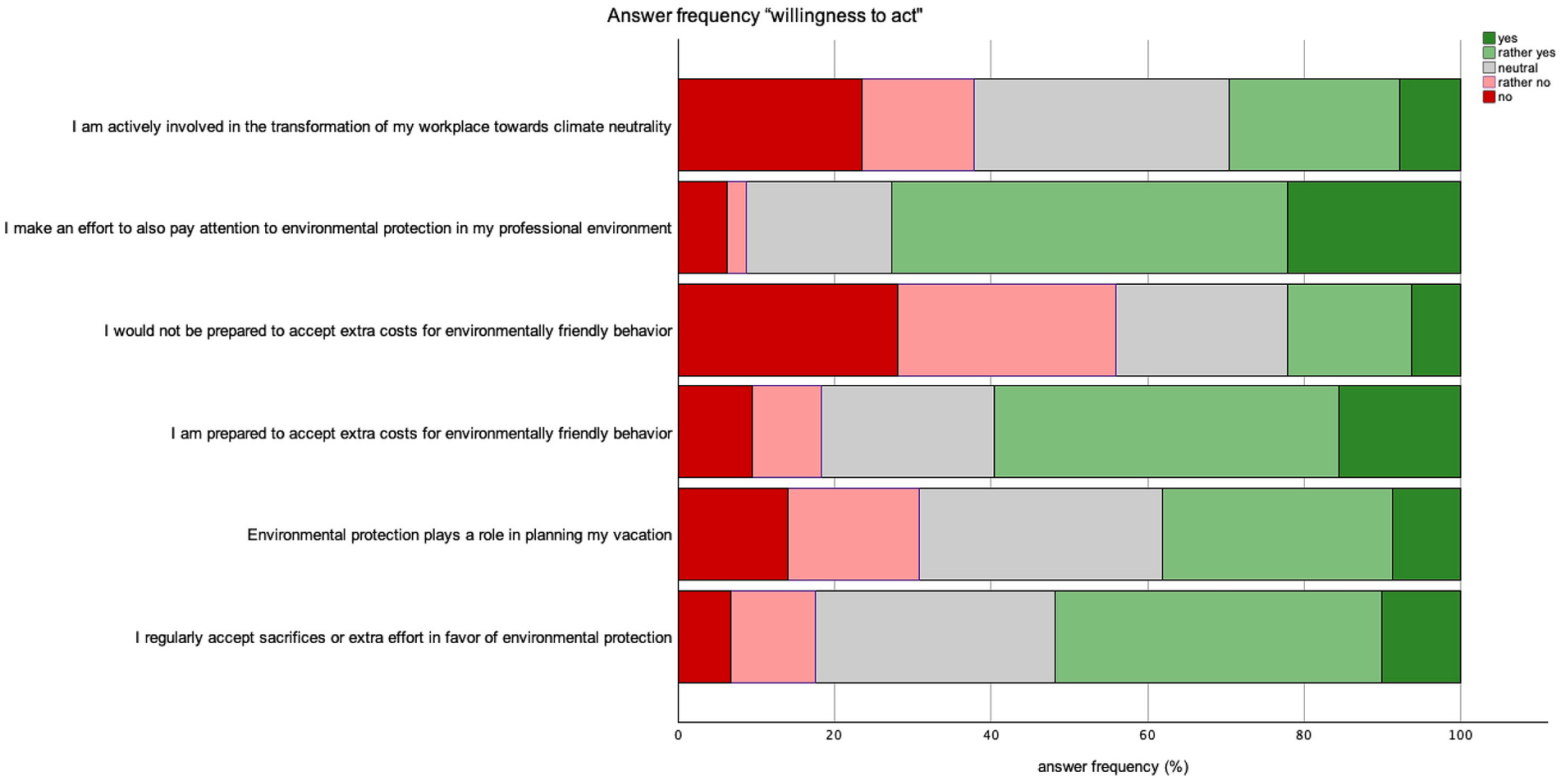
Answer frequency “willingness to act”.

66.9% of participants (*n* = 267) stated that they pay attention to environmental protection on a daily basis. Whereas 2% (*n* = 8) of patients indicated never paying attention to environmental measures, aligning with previous responses. The frequency of responses in absolute numbers is depicted in Figure 4. Participants’ willingness to protect the environment was assessed based on six statements comprising actionable items and statements about environmental protection. The frequency of responses is depicted in Figure 5. Additionally, we used the responses to establish a scale of respondents’ readiness to act, demonstrating sufficient intervariable consistency and reliability using Cronbach’s alpha (0.773). The resulting scale revealed a mean willingness to act of 3.33 on a scale of one to five and was utilized in further regression analysis.

**Figure 6:**
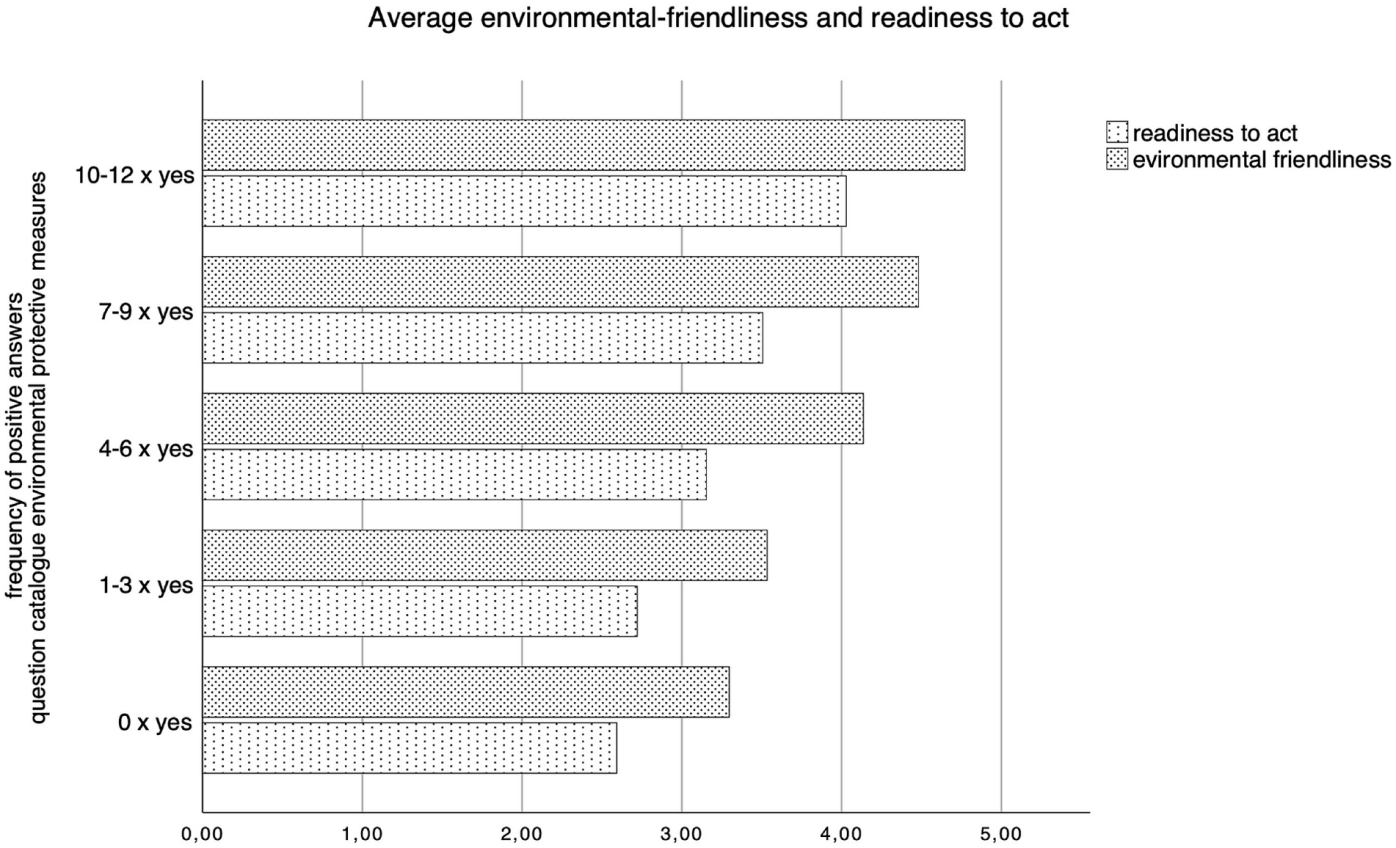
Average environmental-friendliness and readiness to act by positive answer frequency.

To validate the scales of environmental friendliness and readiness to act, respondents were grouped by the frequency of positive answers toward environmental protection measures. A higher frequency of “yes” responses was associated with higher scores in environmental friendliness and readiness to act. There was a significant positive correlation between readiness to act and environmental friendliness (Pearson Correlation 0.685, *p* < 0.001). An increasing number of climate actions respondents took positively correlated with environmental friendliness (Eta 0.576; *p* < 0.001) and willingness to act (Eta 0.462; *p* < 0.001). The difference in means of willingness to act and environmental friendliness between groups with different answer frequencies was statistically significant. Figure 6 depicts the average environmental-friendliness and readiness to act, as indicated by the frequency of positive responses.

### 3.4 CO_2_ neutrality in clinical care

While 25.8% of respondents remained impartial to the question, 55.9% (*n* = 223) stated that they were not informed about the climate impact of the healthcare system. Concurrently, 52.3% (*n* = 209) of participants believed that CO_2_-neutral practices already have a place in hospitals. Approximately half of the respondents (51.1%, *n* = 204) indicated a desire for more information about CO_2_ emissions in the healthcare sector, while 48.9% (*n* = 195) expressed no interest in further information. There was no statistically significant difference between these groups regarding age and subjective degree of illness. However, the mean values of environmental friendliness and readiness to act were significantly higher in the group seeking more information (Whitney *U* Test, *p* < 0.001). Seventy-three patients (18.3%) reported being informed about hospitals’ climate impact. No statistically significant difference in answer frequency concerning knowledge about CO_2_ emissions in healthcare among age groups was observed.

### 3.5 Attitude toward environmentally friendly alternatives in healthcare

The majority of respondents (75.2%; *n* = 300) expressed a favorable attitude toward a study on sustainability in operations, while 88.2% (*n* = 525) welcomed environmentally friendly initiatives in hospitals. Furthermore, a significant proportion of respondents (86.5%; *n* = 345) welcomed the use of environment friendly alternatives, provided that the quality standard in the operating theater remained unchanged.

Participants were asked if they would like to be directly confronted with the carbon footprint caused by their medical treatment, using a system similar to the Nutri-Score (18) 63.2% (*n* = 252) of respondents expressed the desire to be informed about the climate impact of their treatment by such a system, while 15% (*n* = 60) would rather not. Roughly half of respondents with a positive view toward a grading system concerning medical procedures (54.4%, *n* = 137) indicated that it would influence their choice of treatment, while 13.5% (*n* = 43) stated it would not.

**Figure 7:**
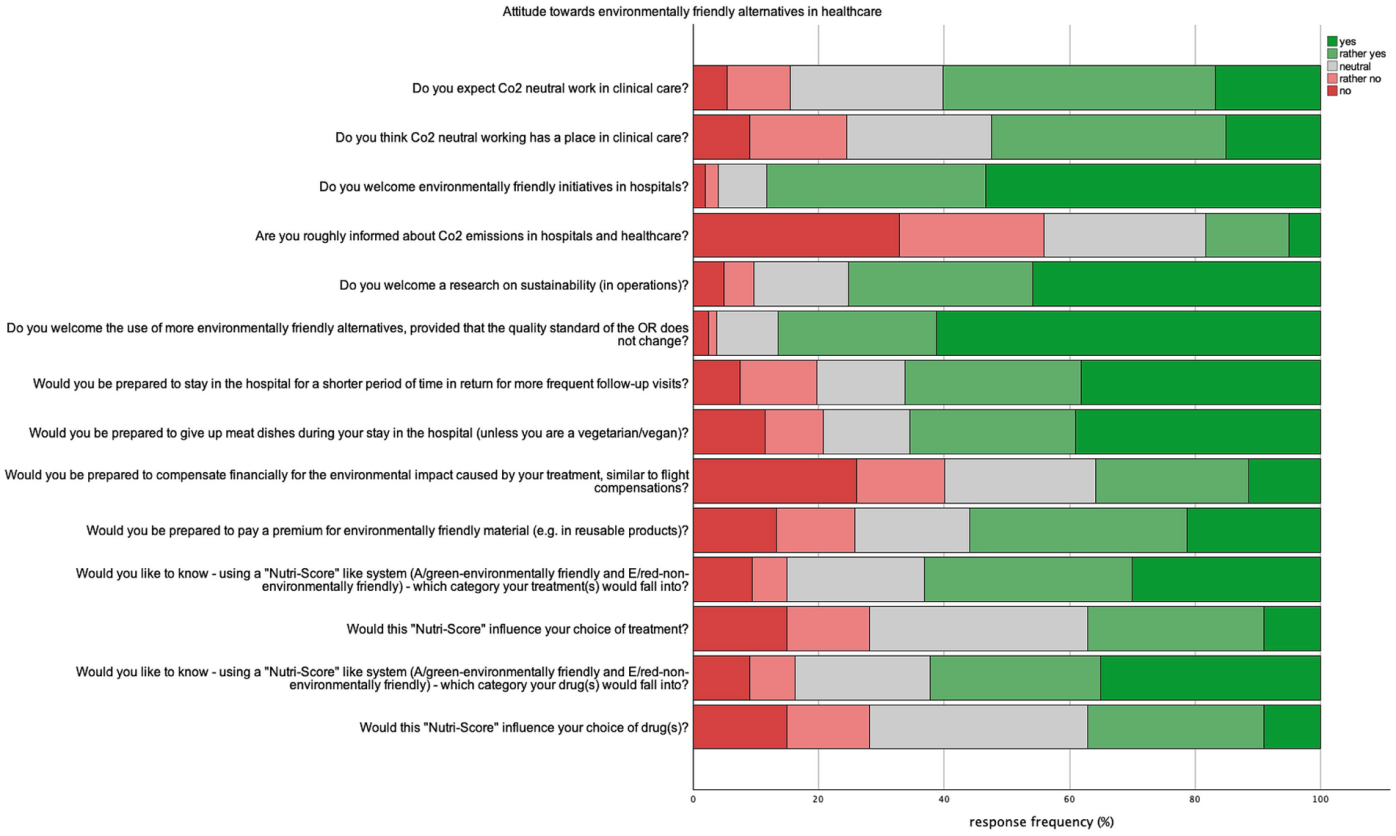
Attitude toward environmentally friendly alternatives in healthcare.

Similarly, 148 respondents (62.2%) expressed a desire to know the environmental impact of their medication using a “Nutri-score” scale, while 16.3% (*n* = 65) would rather not know. Of these respondents with a positive attitude toward a scaling system, 65% (*n* = 161) would allow the choice of their medication to be influenced by this score, while 12.1% (*n* = 30) would not. Notably, 89.9% (*n* = 133) of all respondents who would let their choice of treatment be influenced by its environmental friendliness would also choose their medication according to its environmental impact. A subgroup analysis revealed that neither the current use of medication, age, nor education level significantly influenced attitudes toward a Nutri-Score-like system or the likelihood of medication choice being affected by it. Overall, a significant portion of patients expressed a willingness toward environmental protective actions. 66.2% (*n* = 264) would be willing to stay in the hospital for a shorter duration and attend follow-up visits more frequently. Conversely, 35.8% of respondents (*n* = 143) were prepared to pay compensation for CO_2_ emissions of their procedure, similar to CO_2_ compensation for air travel, while 40.1% (*n* = 160) were not willing to pay such compensation. 81% of the respondents were not willing to pay more into health insurance to support environmentally friendly alternatives. Figure 7 illustrates respondents’ attitudes toward environmentally friendly alternatives in healthcare, with answer frequencies shown in percentages.

Under the condition that safety and quality standards are not affected, respondents were asked to choose from a list of items which ones they would like to see implemented in the future. The results are summarized in Table 5.

**Table 5:** Respondents were asked to select preferred future implementations from a provided list, under the condition that safety and quality standards remain unaffected.

Patient’s perspective on future developments		Count (%)
I would like to see environmentally friendly alternatives in healthcare	Yes	279 (69.9)
	No	120 (30.1)
I would like more information about CO_2_ emissions from healthcare	Yes	204 (51.1)
	No	195 (48.9)
I would like to maintain the current status quo	Yes	52 (13.0)
	No	347 (87.0)
I am not interested in environmentally friendly alternatives in healthcare	Yes	40 (10.0)
	No	359 (90.0)
I would be prepared to pay more into health insurance to support environmentally friendly alternatives	Yes	74 (18.5)
	No	325 (81.5)
I see it as the state’s obligation to legally establish environmentally friendly alternatives.	Yes	211 (52.9)
	No	188 (47.1)

The frequencies of selected items are presented in both absolute numbers and as percentages of the total cohort.

### 3.6 Further analysis

Regression analyses revealed that having children has a significant positive effect on environmental awareness (*β* = −0.112, *p* = 0.05). Additionally, older age groups are associated with higher values in environmental friendliness (*β* = 0.132, *p* = 0.024). However, the degree of illness, gender, relationship status, and education had no significant effect. The overall model was statistically significant (*F* = 3.405, *p* = 0.003). Consistency and reliability measures were conducted as described in the Methods section.

## 4 Discussion

There is a vast majority of evidence toward the effect of global warming on public health (7). However there still seems to be a lack of strong initiatives to reduce the carbon footprint within the health care sector. Within our first Study we tried to evaluate what the health care worker is willing to do (18). Within our work we could show that there is limited awareness and information about carbon footprints in surgical departments in German hospitals. Nevertheless, the majority of surgeons across all age groups are willing to acquire new insights and adapt to changes in order to reduce energy consumption and carbon dioxide production. This is in accordance with the findings within the UK and Ireland (19). Within this study we delved into patients’ perspectives on climate change and environmental sustainability within the healthcare sector, uncovering several noteworthy findings.

Patients in our cohort generally exhibit environmental awareness and recognize the impact of climate change on health. Most prioritize climate-friendly practices in their personal lives, consistent with large survey studies from Germany and the UK (20, 21). However, a significant proportion of respondents are unaware of the healthcare system’s CO_2_ emissions and their impact on climate change. Many patients assume that environmental protection measures are already in place within hospitals (Figure 7). This suggests that hospitals should prioritize educational campaigns to raise awareness about the carbon footprint of healthcare, potentially increasing patient engagement in sustainability efforts. This can be done through incorporating educational materials into patient interactions, both in-person and through digital platforms. Hospitals can also host workshops, provide sustainability tips, and involve patients in green initiatives such as telemedicine. These efforts, supported by ongoing campaigns and feedback loops, could help foster a culture of environmental responsibility within the healthcare setting.

Analyzing our cohort, we observed a notable gender distribution difference, with a higher proportion of male respondents. However, cross-sectional surveys from the USA and Canada indicate that, after adjusting for scientific knowledge, there are no significant gender differences in environmental attitudes. This aligns with our findings (22, 23). Our survey predominantly included respondents over 50 years old, with a notable peak in the 25–35 age group. Despite the significant representation of older individuals in our cohort, they are often underrepresented in public climate change discourse. We found no significant differences between age groups regarding environmental protective attitudes and actions. While some studies suggest older adults are less concerned about climate change and prefer public funds to address other global challenges, other studies found that regarding environmental health and anticipated health impact of climate change, there is a comparable level of concern across all age groups (24–26). In light of impending demographic shifts, the interest and knowledge of the baby boomer generation regarding climate-protective measures are of particular concern, as they will constitute an increasingly significant portion of the patient population in the coming years. Our research demonstrates that engagement in climate action is not limited by age; there is significant interest among both older and younger generations, underscoring the need for all age groups to be included in climate discourse. The findings from our initial study reinforce this, as both senior and junior surgeons express a strong enthusiasm for contributing to climate protection efforts (18). Several studies report a positive correlation between the degree of education and environmental behavior. However, our data did not show a significant correlation in this context, though these data points were not collected in depth (27).

In our cohort, the majority of patients had a surgical procedure planned during their stay regardless of emitting department. This underscores the importance of discussing the impact of surgical departments on healthcare’s carbon dioxide emissions. Notably, the surgical area is expected to offer the largest potential savings in carbon emissions within the hospital setting (28). This begs the question of whether patients will endorse changes in these areas. The findings of our survey indicate a notable patient inclination toward environmentally sustainable practices within the healthcare domain. The broader adoption of climate-friendly alternatives in operating rooms garners support, contingent upon the maintenance of established standards in patient care and treatment protocols (Figure 7). In perioperative care measures like reduced length of stay and optimizing nutrition present viable options for carbon footprint reduction. When it comes to the operating room, multiple changes can be adopted to decrease carbon emissions. Using less volatile anesthetic gasses, upgrading ventilation systems, decrease the use of packaging and single use devices as well as optimize supply chains and endorse environmental preferable purchasing (EPP) (3, 9, 29–31).

Within the framework of this survey, particular significance is attributed to interventions directly affecting the hospital experience of patients and requiring their cooperation. 47.1% of Patients in our cohort stayed at the hospital 1 to 5 days and 31.1% 5–10 days. To give an example: An acute care unit produces 5.5 kg of solid waste and emits 45 kg of CO_2_-equivalents per day of hospitalization. The primary sources of emissions stem from the procurement of consumable goods, building energy utilization, acquisition of capital equipment, food services, and staff commuting (32). Reducing the duration of inpatient stays can effectively mitigate CO_2_ emissions, with particularly significant reductions achievable through same-day surgery procedures (33). In the context of cholecystectomies, same-day discharge presents as a viable and safe strategy for carbon emission reduction. However, further investigations are necessary to confirm its efficacy. An argument can be posited for the feasibility of implementing this approach, particularly for laparoscopic procedures in carefully selected patients with minimal comorbidities, aiming to reduce hospitalization duration on a broader scale. The endorsement of this option to healthcare providers and policymakers is seemingly straightforward, given the concurrent benefits of cost and resource savings (adjusted for severity of admitting diagnoses and outcome measures) (34–37). Our findings suggest that patients are receptive to shorter hospital stays and demonstrate willingness to attend follow-up appointments more frequently (Figure 7). Thus, optimizing the length of hospital stays following minimally invasive surgery and developing protocols for safe postoperative follow-up would be a practical initial step for surgical departments to reduce both costs and carbon emissions. Additionally, this approach offers significant benefits for patients, including time savings and reduced travel costs, particularly in complex procedures where treatment at specialized centers is required.

Hospital food presents another relatively straightforward yet high-impact opportunity for carbon reduction. Transitioning to plant-based diets in hospital settings not only contributes to sustainability but also offers significant health benefits, such as reducing risk factors for diabetes, cardiovascular risk factors and circumventing the increased cancer risks associated with red meat consumption. Plant-based diets can potentially lower greenhouse gas emissions by up to 49% (38–41). A study from the UK corroborates our findings, indicating that the majority of patients are willing to replace meat with plant-based options in hospital meals (42). Our survey results suggest that patients are generally open to adopting a modified hospital diet. As illustrated in Figure 7, the majority of respondents expressed willingness to forgo meat dishes during their hospital stay. While it is often argued that dietary changes must accommodate all patients, our findings indicate that many patients are receptive to vegetarian options. To implement this, hospitals could introduce flexible meal plans that prioritize plant-based options while ensuring adequate nutritional balance, allowing for patient preferences and dietary needs to be met.

Another approach to reducing the carbon footprint across industries is Environmentally Preferable Purchasing (EPP). Similar programs exist in the USA and Europe. EPP involves procuring products and services that have a reduced impact on human health and the environment compared to other products and services serving the same purpose. This practice considers factors such as the sustainability of raw materials, energy efficiency, recyclability, reduced toxicity, and overall environmental footprint throughout the product’s lifecycle, from production and use to disposal. EPP aims to promote more sustainable consumption and encourage the development and use of greener products and services. EPP can be applied to account for CO_2_ emissions of products used in surgery, medications, prosthetics, bandage materials, and more (9, 43, 44). We investigated patient preferences regarding the disclosure of the environmental impact of their surgeries or medications using a system akin to the Nutri-Score. The majority of respondents favored receiving such information; however, fewer were inclined to allow this information to influence their treatment choices. Hakonsen et al. (45) conducted an online survey in Sweden, where participants chose between more effective treatments and environmentally friendly options for conditions ranging from the common cold to stroke. The study revealed that for mild conditions, participants predominantly opted for environmentally friendly treatments, whereas for severe conditions, the more effective treatments were preferred (45). This suggests that as personal risk increases, the adoption of EPP diminishes. Environmentally Preferable Purchasing (EPP) can play a pivotal role in reducing healthcare’s environmental footprint; however, its success will hinge on striking a careful balance between sustainability and patient care priorities, particularly in high-risk clinical settings. A system similar to the Nutri-Score could be utilized to communicate EPP initiatives to patients, fostering transparency and understanding of how sustainable choices are integrated without compromising care.

Our findings, as shown in Figure 7, suggest that patients find the Nutri-Score concept appealing, as it raises awareness and promotes education rather than influencing treatment decisions directly. Similarly, for any sustainability measures, it is critical to ensure patient safety and clearly communicate that quality of care remains uncompromised. This underscores the core objective of our study: patients must be reassured that these initiatives will not negatively impact the quality of their care.

Patients generally express an interest in climate-friendly practices in the healthcare sector, reporting environmental consciousness and engagement in protective measures in their private lives. However, the question remains whether these attitudes translate into action. When asked about the environmental protection measures they engage in daily, it was observed that the level of intervention chosen was inversely related to factors such as time, financial burden, and personal comfort. Notably, respondents were less enthusiastic about actions requiring financial compensation, consistent with findings in behavioral economics. A study from Germany by Venghaus et al. (46) describes this attitude-behavior gap: Despite broad, positive attitudes toward climate protection and high awareness of climate change issues, these attitudes do not readily translate into significant behavioral changes (47). Farjam et al. (48) found that environmental attitudes influenced behavior only in low-cost situations, supporting the low-cost hypothesis of environmental behavior. This hypothesis suggests that individuals concerned about the environment will undertake low-cost actions to reduce cognitive dissonance between their attitudes and the rational understanding of their environmental impact but avoid higher-cost actions despite their greater potential for environmental protection (46). In a German healthcare cohort, increasing financial compensation can be seen as biased. Rising healthcare costs and the impending retirement of the boomer generation will further strain German health insurance systems, ultimately affecting contributors (48). Hence, it is understandable that insured individuals are less willing to pay additional premiums on their health insurance bills. In light of this, the healthcare sector must prioritize interventions that align with patients’ willingness to engage in low-cost, low-effort environmental actions. For example, changes such as optimizing hospital processes, reducing waste, or adjusting procurement policies could offer significant environmental benefits without directly burdening patients. Additionally, healthcare systems should consider integrating sustainability measures into existing protocols without transferring additional costs to patients, as financial barriers could hinder widespread adoption.

In conclusion, the question remains: who is responsible for driving the shift toward greener healthcare? Respondents in our cohort predominantly view the state as the key agent of change. Research generally identifies three primary actors: the market, regulators, and individuals. Effective environmental protection necessitates a synergistic approach involving government regulations, individual actions, and market-based solutions. The state plays a crucial role in establishing the legal and institutional framework, individuals contribute through personal responsibility and advocacy, and the market drives innovation and efficient resource management within the regulatory context. Additionally, cost reductions achieved through environmentally friendly measures present a compelling argument for market-driven change (49–51).

This study has several limitations. The use of purposive sampling and voluntary participation may have introduced selection bias, as those more interested in environmental issues may have been overrepresented. The online survey format could have excluded patients with limited access to technology, particularly older or socioeconomically disadvantaged individuals, limiting generalizability. Additionally, the cross-sectional design captures attitudes at a single point in time, precluding the ability to assess changes or causal relationships. The reliance on self-reported data raises the possibility of social desirability bias. Lastly, while environmental awareness was high, the gap between awareness and tangible action remains unclear and warrants further exploration.

## 5 Conclusion

Our study reveals significant patient awareness and concern for climate change, with strong support for sustainable practices in healthcare. Yet many patients lack understanding of the healthcare sector’s carbon footprint, highlighting the need for better education. Patients are generally open to environmentally friendly changes, particularly in surgical departments, hospital food, and procurement, as long as care quality is maintained. However, practical engagement decreases when costs or personal discomfort are involved. A coordinated approach—encompassing policy changes, market innovations, and patient education—is crucial to reducing healthcare’s carbon footprint. Further research should focus on bridging the gap between environmental awareness and actionable behaviors in healthcare settings.

## Data Availability

The raw data supporting the conclusions of this article will be made available by the authors, without undue reservation.

## Supplementary material

Supplementary Data Sheet 1 Survey questions.
