# Supplementary material for: Focus on patient perspectives in climate action policies for healthcare. A German survey analysis on what patients are willing to do
Source: Front Public Health. 2024 Nov 26;12:1477313. doi: 10.3389/fpubh.2024.1477313 (PMC11629199; doi:10.3389/fpubh.2024.1477313)
Supplement: Supplementary Data Sheet 1 — Survey questions. [file Data_Sheet_1.PDF]

LimeSurvey

Dear patients, welcome to our survey "Sustainability in the hospital". Thank you very much for helping us with your answers. We kindly ask you to fill out everything as best as possible. We want to emphasize that there are no right or wrong answers. Ready? Let's go...

Section A: Personal information

A1. Please state your sex

- male ☐
- female ☐
- divers ☐

A2. Please state your age

- 18-25 ☐
- 26-35 ☐
- 36-45 ☐
- 46-55 ☐
- 56-65 ☐
- 66-75 ☐
- 76-85 ☐
- <85 ☐

A3. Please state your relationship status

- single and not living in a partnership ☐
- single but living in a partnership ☐
- married/registered partnership ☐
- widowed ☐
- divorced ☐

A4. Do you have children?

- yes ☐
- no ☐

LimeSurvey

A5. What qualifications do you hold?

- [ ] Certificate of Secondary education (CSE) or equivalent
- [ ] General certificate of Secondary education (GCSE) or equivalent
- [ ] Advanced technical college entrance qualification
- [ ] A levels
- [ ] other qualifications such as apprenticeship or similar
- [ ] University of applied sciences
- [ ] University (bachelor, master degree)
- [ ] Promotion/ doctorate
- [ ] PhD/ postdoctoral lecture qualification
- [ ] No qualifications

A6. Please state your place of residence (city, state)

A7. What kind of stay do you have in this hospital?

- [ ] out-patient ("I'm neither staying the night before nor after the intervention in the hospital")
- [ ] in-patient ("I'm staying at least one night in the hospital")

A8. Please mark in which department you are being treated

- [ ] Internal medicine (cardiology, gastroenterology, hepatology, nephrology, endokrinology, neurology, rheumatology, haematology, oncology, angiology)
- [ ] Trauma surgery and orthopedics
- [ ] General, visceral and transplant surgery
- [ ] Neuro surgery
- [ ] Urology
- [ ] OB/GYN
- [ ] Ear-Nose-Throat (ENT) department
- [ ] Plastic/ reconstructive surgery
- [ ] Other

Other

A9. On a rising scale from 1-5: How sick would you consider yourself? (1= slightly sick; 5=severly sick)

- [ ] 1
- [ ] 2
- [ ] 3
- [ ] 4
- [ ] 5

A10. Please state on the basis of a scale to what extent you have pre-existing conditions. 1=no pre-existing condition(s); 2= slight pre-existing condition(s); 3=moderate pre-existing condition(s); 4=rather severe pre-existing condition(s); 5= severe pre-existing condition(s)

- [ ] 1
- [ ] 2
- [ ] 3
- [ ] 4
- [ ] 5

A11. Please check which level applies to you.

- Normal, no complaints, no evidence of disease ☐
- Able to carry on normal activity, minor signs or symptoms of disease ☐
- Normale activity with effort, some signs or symptoms of disease ☐
- Care for myself, unable to carry on normal activity or to do active work ☐
- Require occasional assistance, but able to care for most of my personal needs ☐
- Require considerable assistance and frequent medical care ☐
- Disabled, require special care and assistance ☐
- Severely disabled, hospital admission is required ☐

A12. Is an operating procedure planned? Has an operating procedure already been carried out?

- No ☐
- Yes, it's planned ☐
- Yes, it has already been carried out ☐

A13. Are you scheduled for treatment of a malignant disease? Are you currently undergoing treatment for a malignant disease?

	Yes	Uncertain	No
	☐	☐	☐
	☐	☐	☐
	☐	☐	☐

A14. Please state if you regulary take medication(s)- if so how many?

- No medication ☐
- less than 5 drugs ☐
- more than 5 drugs ☐
- more than 10 drugs ☐

A15. How long do you think you will be staying at the hospital?

- 1-5 days ☐
- 5-10 days ☐
- 10-15 days ☐
- longer than 15 days ☐

LimeSurvey

A16. Which diet are you currently on?

- Meat eater ☐
- Vegetarian ☐
- Vegan ☐
- Flexitarian (mostly abdictation of meat, exeptions on holidays etc.) ☐
- Other (e.g. fruitarian, pescetarian etc.) ☐

Section B: Environmental information

B1. Please evaluate the following statements

	Yes	Rather yes	neutral	Rather no	No
I care about the environment (in general)	☐	☐	☐	☐	☐
I am aware of the climate crisis of our time	☐	☐	☐	☐	☐
Environmental friendly behaviour is of high value in my private life	☐	☐	☐	☐	☐
Environmental friendly behaviour is of low value in my private life	☐	☐	☐	☐	☐
Are you taking measures in your private life in favour of the environment and its protection?	☐	☐	☐	☐	☐
Are you taking (regularly or on occasion) part in conversations about climate crises related topics?	☐	☐	☐	☐	☐

B2. Which measure are you taking? (multiple choice possible)

- Waste separation ☐
- Buying groceries of controlled ecological cultivation ☐
- Avoiding waste (f.e. "unpacked shops", buying groceries on the market with reusable bag or similar) ☐
- Using CO2-neutral transportation (going by bicycle, public transportation, electric car etc.) ☐
- Saving water (e.g. turning off water while brushing your teeth) ☐
- Taking showers instead of taking a bath ☐
- Avoid using batteries/ Using devices with grid operation or batterie-free devices instead ☐
- Air-drying your hair instead of using a blow-dryer ☐
- Avoid using "coffe-to-go" cups/ Using your own cup to refill ☐
- Avoiding meat ☐
- Avoiding animal-based products ☐
- Buying groceries from controlled organic cultivation ☐

LimeSurvey

No measures ☐

Other ☐

Other

B3. **How often do you pay attention to environmental protection in your everyday life?**

- Daily ☐
- Weekly ☐
- Monthly ☐
- Occasionally ☐
- Never ☐

B4. **Please decide on the following statements whether these statements apply to you or not**

	Agree	Rather agree	neutral	Rather disagree	Disagree
I accept regularly waiver or extra expenses in favour of environmental protection	☐	☐	☐	☐	☐
Environmental protection plays a role when planning my holiday	☐	☐	☐	☐	☐
I am willing to incure additional cost for an environmental-friendly behaviour	☐	☐	☐	☐	☐
I am not willing to incure additional cost for an environmental-friendly behaviour	☐	☐	☐	☐	☐
I am taking care to pay attention to environment protection in my professional surroundings	☐	☐	☐	☐	☐
I am actively involved in redesigning my workplace towards climate neutrality	☐	☐	☐	☐	☐

Section C: Environmentalism in clinical care

C1.

	Stronlgy agree	Rather agree	neutral	Rather disagree	Disagree
Do you think CO2-neutral working is generally of particular importance to the clinical care right now	☐	☐	☐	☐	☐
Do you expect CO2-neutral working to be of particular importance to the clinical care	☐	☐	☐	☐	☐
Do you welcome environmental-friendly initiatives in hospitals	☐	☐	☐	☐	☐

LimeSurvey

	Stronlgy agree	Rather agree	neutral	Rather disagree	Disagree
Are you roughly informed about the CO2-emissions in hospitals or health care	☐	☐	☐	☐	☐
Do you welcome research on the subject sustainability in the operating theatre	☐	☐	☐	☐	☐
Do you welcome the use of eco-friendly alternatives without changing the quality of the operating procedure	☐	☐	☐	☐	☐
Would you be willing to shorten your hospital stay and come in more frequently for follow-ups	☐	☐	☐	☐	☐
Would you be willing to abstain from meat dishes during your hospital stay	☐	☐	☐	☐	☐
Would you be willing to financially compensate the environmental pollution that resulted of your treatment	☐	☐	☐	☐	☐
Would you be willing to pay extra compensation for environmental friendly material (e.g. reusable products)?	☐	☐	☐	☐	☐
Would you want to know- on the basis of a "nutri-score" similar tool (A/green=environmental friendly; E/red= not environment friendly)- in which category your treatment would be classified	☐	☐	☐	☐	☐
Would you want to know- on the basis of a "nutri-score" similar tool (A/green=environmental friendly; E/red= not environment friendly)- in which category your medicine would be classified	☐	☐	☐	☐	☐
Would this "nutri-score" similar tool influence your choice of treatment	☐	☐	☐	☐	☐
Would this "nutri-score" similar tool influence your choice of medicine	☐	☐	☐	☐	☐

C2. For the future: Please mark in the following what you would wish for in the future- under the condition that the security and quality of the operating procedures would remain the same. (Multiple choice possible)

- I wish for environmental friendly alternatives in clinical/health care ☐
- I wish to get more information about the CO2 emissions of health care ☐
- I wish to maintain the current state ☐
- I am not interested in environmental friendly alternatives in health care ☐
- I would be willing to pay more into health insurance in order to support environmental friendly alternatives ☐
- I think it is the government's obligation to offer environmental friendly alternatives ☐
- Other ☐

Other

[ ]

Section D: Final statements

D1. For the final question we have some entertaining facts for you. Would you have known this? Maybe suspected? Or does it suprise you?

	Yes	Uncertain	No
Are you aware that health care- if you were to consider it as country- ranks fifth in the world in terms of CO2-emissions?	☐	☐	☐
Are you aware that the healthcare sector, especially the pharmaceutical sector, produces more CO2 emissions worldwide than the automotive industry?	☐	☐	☐
Are you aware that more patients are to be expected due to climate change?	☐	☐	☐

Thank you very much for participating in our survey "Sustainability in the hospital". We hope you liked it.
